# Enhanced distillate production of stepped solar still via integration with multi-stage membrane distillation

**DOI:** 10.1038/s41598-025-95098-4

**Published:** 2025-04-12

**Authors:** A. A. Mahgoub, S. M. Elsherbiny, O. A. A. El-Masry, O. A. Elsamni

**Affiliations:** Mechanical Engineering Department, Faculty of Engineering, Alexandria University, P.O. Box 21544, El-Chatby, Egypt

**Keywords:** Multi-basin, Stepped solar still, Passive solar desalination, Membrane distillation, Distillate enhancement, Compact system, Numerical analysis, Solar thermal energy, Thermodynamics, Environmental sciences, Engineering, Mathematics and computing, Sustainability, Solar energy

## Abstract

Arid and coastal regions, such as Egypt, face significant challenges in meeting potable water demands driven by population growth and sustainable development. Energy-efficient desalination technologies are urgently needed to reduce reliance on conventional energy sources. This study introduces a novel integration of a thermal membrane with a stepped solar still, leveraging basin heat to drive membrane distillation (MD). The system employs multiple membrane layers and water–vapor absorbent wicks to capture vapor and transfer latent heat, enabling cascading preheating of brine layers. A validated mathematical model assessed the system’s performance under varying conditions, accommodating up to ten stages. Results reveal that increasing MD stages significantly enhances productivity, achieving a peak daily output of 47 L/m^2^ day—five times that of a passive multi-basin solar still. However, productivity gains plateau beyond three stages, indicating an optimal balance between complexity and efficiency. The system’s performance is affected by ambient and seasonal conditions, including wind speed and temperature fluctuations observed during trials in June, May, and December, requiring additional modifications for stabilization. This study demonstrates the potential of integrating MD with solar stills as a scalable, energy-efficient desalination solution for arid regions. Future research should focus on optimizing stage configurations and evaluating economic feasibility for large-scale applications.

## Introduction

Freshwater scarcity is a growing issue due to population growth and industrial expansion, especially in remote, arid coastal areas. Solar stills, which mimic the natural water cycle, use solar energy to purify water, offer a cost-effective solution for moderate freshwater production^1^. They consist of a salinized water basin, solar absorber plate, glass cover, distillate trough, and insulation. They are simple, low-cost, and effective at removing contaminants. The potential economic market of solar stills includes rural and remote places, tiny islands, resorts, coastal communities, and regions with brackish water ground wells and bore wells for solar stills^2^.

Before the development of RO systems, there were several large-scale basin solar stills projects that have been installed and successfully operated. Starting from the smaller size, Rubio et al.^3^ carried out evaluation studies for solar still areas ranging between 54 and 384 m^2^. Delyannis and Delyannis^4^ carried out an economical study for a 1400 m^2^ in Portugal and concluded that the solar stills remain the optimum solution for small communities in arid areas despite the high initial cost. In India, Natu et al.^5^ investigated the production rates of 1867 m^2^ solar stills and could obtained a correlation to relate the production rate with solar intensity, air temperature, and wind velocity. Hirschmann^6^ reported a 4752 m^2^ solar stills that was originally designed to provide the fresh water for miners in coastal area of Chile. In a more recent study, Hota et al.^7^, concluded that a large-scale basin solar still of 10,000 m^2^ area was relatively economical than solar photovoltaic (PV)-powered reverse osmosis units in California, USA.

Solar stills can produce pure water for various uses and come in passive (using only solar energy) and active (requiring extra sources of energy) varieties^8^. Several modifications to the still design and operating conditions have been made to passive stills in order to improve their productivity. The design modifications include; multi-stepped multi basin^9^, multiple slope stills^10^, basin improvements with black coatings^11^, using internal fins^12,13^, conical and hemispherical shape^14^, and condenser optimizations^15^. Operational improvements include water depth control^16^, augmenting the heating process using; external solar water heater^17^; internal reflectors^18^; or using external reflectors^19^, and tilt angle adjustment^20^. Advanced techniques involve nanotechnology and hybrid systems^21^.

Solar stills are shown to be appropriate when the drinking water demand is less than 200 m^3^ per day and local weather conditions are favorable^22^. The problem of the low output of solar stills has spurred academics to develop methods to boost it.

In order to perform a techno-economic comparison in terms of productivity and economic design as well as the efficiency with the goal of commercializing solar still design, Katekar et al.^1^ surveyed different design modifications and carried out a techno-economic comparison to identify the recent effective passive solar stills productivity. They concluded that implementing a multi-basin stepped solar still has several benefits compared to alternative solar still designs. They may be readily expanded and scaled up by including more steps or basins, rendering them appropriate for address water purifying requirements of various scales^23^. They can be integrated with external designs to further improve the distillate productivity rate using external energy sources^24^ or using passive condenser^15^.

The productivity of solar stills has been significantly hindered by inefficiencies in water collection and considerable heat loss from all sides. Ranjan et al.^25^ identified that the basin liner side of solar still assemblies experiences the highest exergy destruction, accounting for 48% of the total solar exergy input. This substantial loss of thermal energy highlights the critical need for effective heat recovery strategies to enhance the efficiency and productivity of solar stills. In response to this challenge, the present study proposes integrating membrane distillation stages at the basin bottom. By harnessing the previously lost thermal energy, the study aims to augment distillate production per unit area, significantly boosting the overall performance and viability of solar still.

Membrane distillation has proven its ability to work at low temperature grades less than $80\;{^\circ }{\text{C}}$ which makes it convenient for conventional solar still basins^26^. The driving force in thermal membrane distillation is the vapor pressure difference across the membrane, which is typically induced by a temperature gradient. This gradient causes water vapor to transfer from the hot feed side to the colder permeate side through a hydrophobic membrane. The process relies on the phase change of water from liquid to vapor, allowing only vapor to pass through the membrane’s pores, effectively separating it from the feed solution. MD operates at lower hydrostatic pressures compared to pressure-driven processes like reverse osmosis, nanofiltration, ultrafiltration, and microfiltration. It also requires less demanding mechanical properties for the membrane and achieves a high rejection factor when separating solutions containing non-volatile solutes, such as salts and colloids. The MD membrane exists in various forms such as sheets, tubes, and bundles of hollow fibers. There are four main configurations by which the water vapor is condensed and collected. They are the direct contact membrane distillation (DCMD), the sweeping gas membrane distillation (SGMD), the vacuum membrane distillation (VMD), and the air-gap membrane distillation (AGMD)^26^.

Membrane distillation has been driven by solar energy as the heating source. For instant, Koschikowski et al.^27^ used a spiral-wound AGMD module fed by hot water from flat plate collectors where the distillate varied from $11\;{\text{L}}/{\text{m}}^{2}$ in winter to $28\;{\text{L}}/{\text{m}}^{2}$ in summer. Similar rates were obtained by Ghim et al.^28^ when using a multi-layer AGMD device made of a polytetrafluoroethylene (PTFE) membrane.

Chiavazzo et al.^29^ developed a passive, high-yielding, low-cost desalination device that uses a multi-stage distillation process to desalinate seawater. The design is based on narrowing the gap between two hydrophilic layers, significantly increasing water vapor permeability. PTFE membranes are inserted between these layers to enhance this effect. The device includes options for single-stage, three-stage, and ten-stage solar distillation, with the three-stage model evaluated in various environments, including laboratory, rooftop, and sea surface conditions. A foam-encased three-stage module, floated on the sea, produced $1.5\;{\text{L}}/{\text{m}}^{2} {\text{h}}$ equivalent to 36 ${\text{L}}/{\text{m}}^{2} {\text{day}}$.

Passive solar desalination has emerged as a promising solution to address global water scarcity, particularly in remote and resource-limited regions. Among the diverse designs, stepped-type solar stills have gained attention due to their higher basin temperatures and enhanced productivity compared to conventional stills. However, despite their advantages, there remains a need for further innovation to improve efficiency and eliminate reliance on expensive materials such as nanoparticles.

In a comprehensive review of novel conceptual design solar systems, Singh^18^ evaluated various passive solar desalination designs and identified Chiavazzo et al.’s^29^ thermal membrane desalination system as the most efficient, both thermally and in terms of distillate productivity. Chiavazzo et al.’s design represents a significant advancement in the field, offering high performance through its innovative use of thermal membrane distillation^29^. However, Singh emphasized that even the most promising designs, including Chiavazzo et al.’s, have room for improvement, particularly in terms of self-sustainability and the elimination of expensive materials such as nanoparticles^18^.

Traditional solar stills suffer from substantial heat losses through the basin bottom, significantly hindering their thermal efficiency and freshwater production. While stepped-type solar stills mitigate these losses by elevating the basin temperature, they still fail to fully utilize the residual thermal energy beneath the basin. A critical gap in the field lies in the absence of an optimized hybrid design that effectively integrates multi-stage membrane distillation with the most efficient solar still configurations.

This study aims to enhance the efficiency of solar stills by developing a novel hybrid system that integrates a stepped solar still with a multi-stage membrane distillation unit positioned beneath the basin. This innovative configuration utilized the wasted heat to augment thermal performance and freshwater yield. The study objectives include the development of a comprehensive mathematical model to analyze system behavior under diverse operating conditions and the optimization of the number of MD stages while ensuring passive system operation. This hybrid design presents a sustainable and high-performance solution for potable water production in arid and coastal regions, particularly suitable for portable and medium-scale applications.

## Description of integrated solar MD distiller

**Fig. 1:**
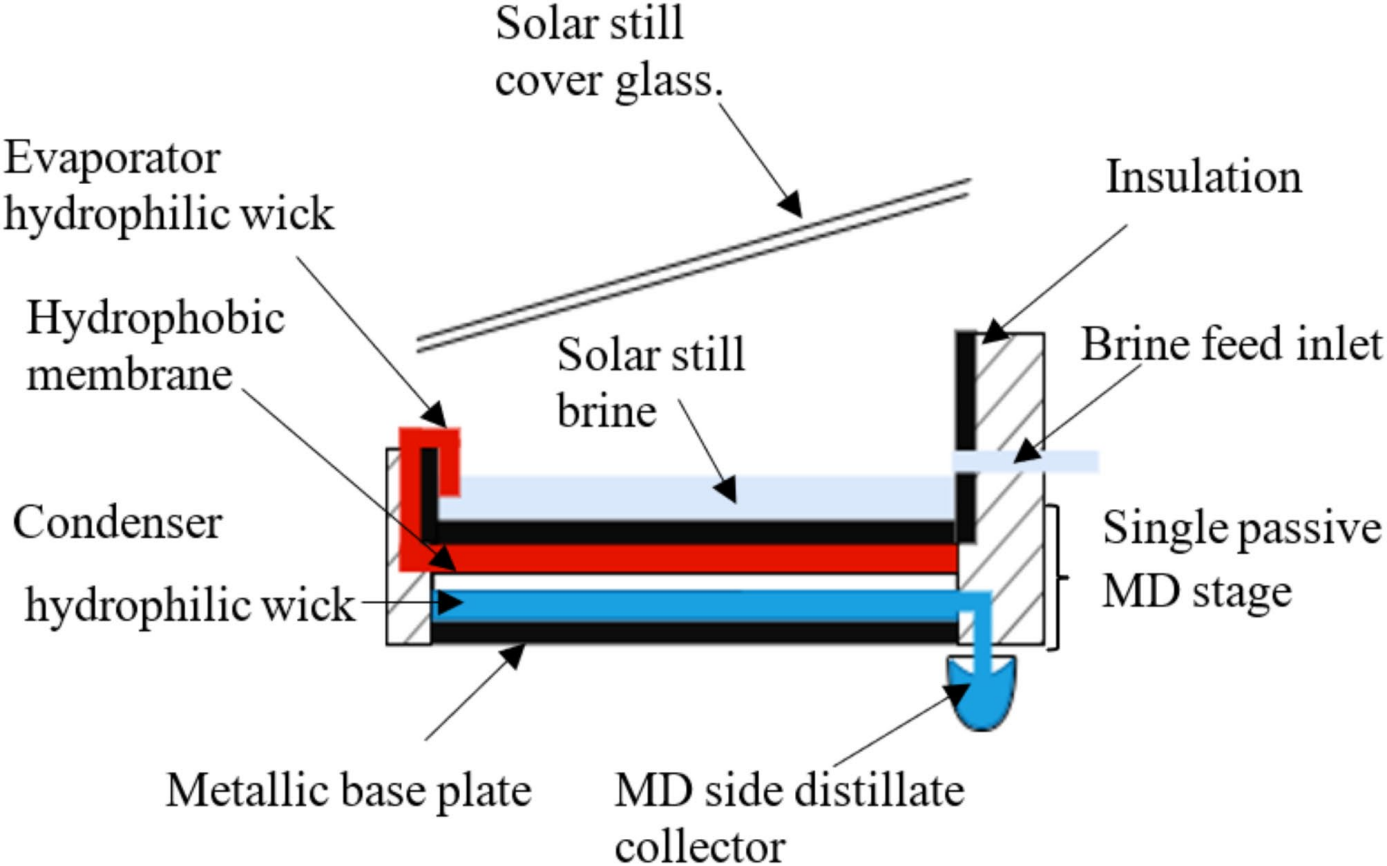
Detailed assembly arrangement of a single basin integrated with a single MD stage with a collector.

The idea of integration, as illustrated in Fig. 1, involves placing the first stage of membrane distillation (MD) beneath the basin of a stepped solar still. A hydrophobic membrane, either in direct contact or with an air gap, separates the brine and distillate. The present design uses high-absorptivity hydrophilic wicks to passively move saline and distilled water without pumps. The passive MD stage comprises three layers:

1. A top hydrophilic wick that absorbs and transports brine from the still basin.
2. A middle hydrophobic PTFE membrane for vapor transfer.
3. A bottom hydrophilic wick that collects water vapor and drains it to the distillate collector.

**Fig. 2:**
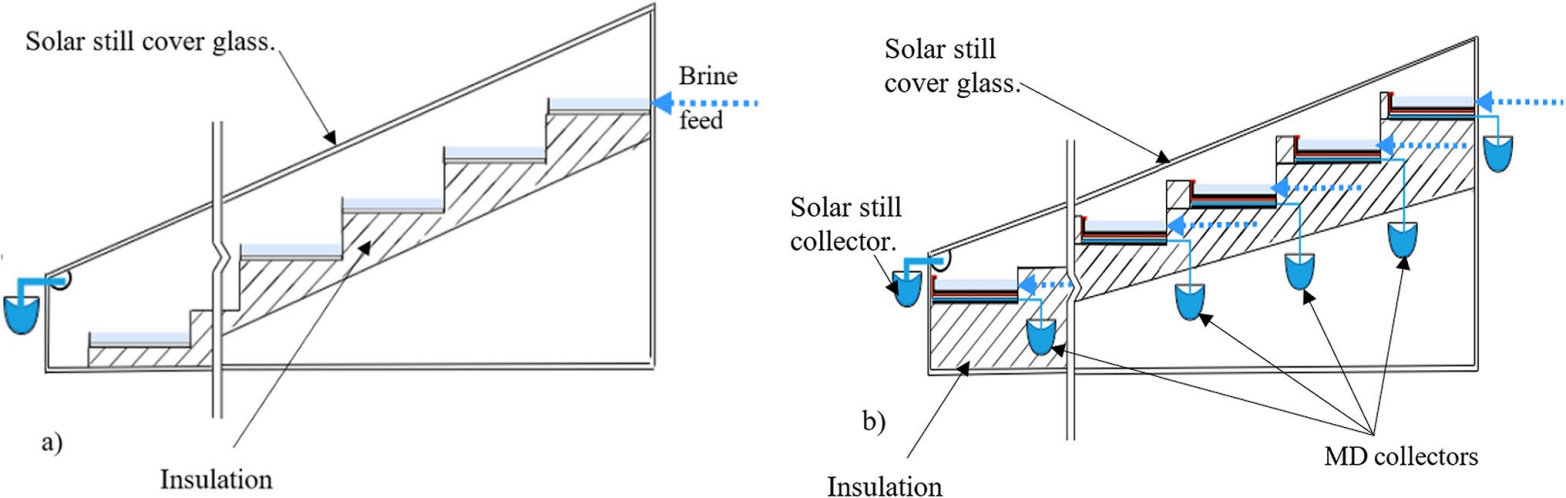
(**a**) Conventional multi basin stepped solar still, (**b**) The proposed integration with single stage membrane distillation.

This wick-based system replaces conventional airgap or direct-contact MDs, promoting efficient flow and distillation. For additional MD stages, a metallic plate can be added below the distillate wick to capture latent heat and transfer it to the subsequent stages. Figure 2 illustrates the modifications made on conventional stepped solar stills to present the way of feeding the seawater and collecting the two types of distillates.

## Methodology

### Mathematical model

A mathematical model of the combined solar still and passive membrane module is developed using energy balance equations and heat transfer principles. Applying the energy balance equations to distinct parts of the solar still leads to the deduction of the water, glass, and the bottom basin temperature in the solar still section. The latter temperature serves as an input to the membrane distillation section where a temperature profile can be deduced after calculating the thermal conduction effect on the membrane module at a given ambient condition. For simplification, kinetic and potential energies are neglected, along with the following considerations:

1. The distillate produced is assumed free of salt.
2. The inner glass surface absorbs the latent heat released by the vapor, fully condensing it into distilled water.
3. Wind flow over the solar still is unidirectional and maintained at a constant speed.
4. The basin absorber is assumed to have negligible reflectivity for incident solar radiation.
5. No duct accumulation, no change in glass properties, and no aging effect of the material with time.
6. The side losses are neglected dure to the thin thickness of the membrane and wicks, following the approach of Chiavazzo et al.^29^.

**Fig. 3:**
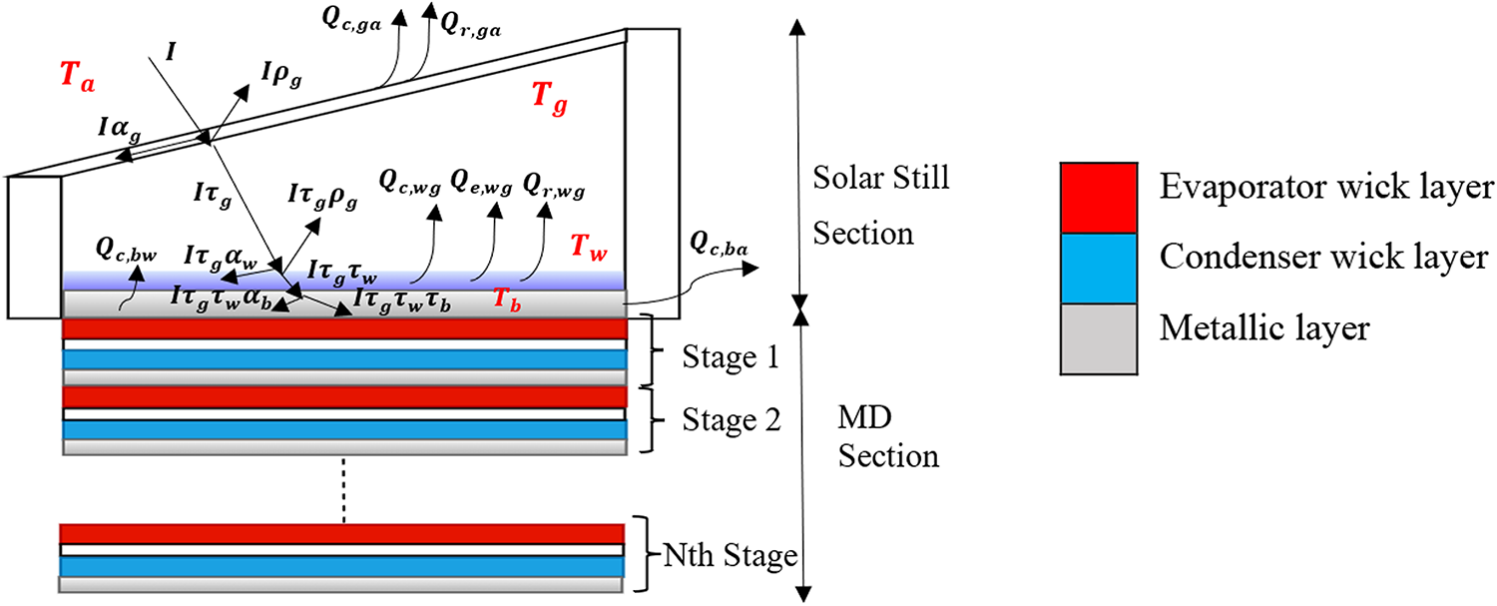
Energy balance on a conventional solar still.

A schematic of the energy balance for main components of the solar still is shown in Fig. 3. Convection, evaporation, and radiation are the three main ways in which heat is transferred inside a solar still. The amount of energy obtained by the water flowing within the solar still and the water input temperature determine how quickly saline water vaporizes.

#### Heat transfer inside the solar still

*a)Convective heat transfer between the water and glass* ($Q_{c,wg}$)*:* Convective heat transfer between the water surface and the inner glass cover occurs due to the temperature difference between them. This process involves free convection in the humid air confined between the water and the glass, with the convective heat transfer rate $\left( {Q_{c,wg} } \right)$ depending on the temperature differential between the water and glass, which can be expressed as in Elsherbiny and Fath^30^ as follows:

1 $$Q_{c,wg} = h_{c,wg} A_{b} \left( {T_{w} - T_{g} } \right)$$

where $h_{c,wg}$ can be obtained from Dwivedi et al.^31^ and Rajamanickam et al.^32^ as follows:

2 $$h_{c,wg} = 0.884{ }[\Delta T]^{\frac{1}{3}}$$

where $\Delta T$ is the temperature difference between the water and the glass which can be expressed in terms of $P_{w}$ and $P_{g}$ (partial pressures of vapor at the water and glass surfaces temperatures, respectively) as follows based on the studies of Dunkle^33^ and Tiwari et al.^34^

3 $$\Delta T = \left( {T_{w} - T_{g} } \right) + \frac{{\left( {P_{w} - P_{g} } \right)\left( {T_{w} + 273.15} \right)}}{{268900 - P_{w} }}$$

4 $$P_{w} = e^{{\left( {25.317 - \frac{5144}{{T_{w} + 273}}} \right)}}$$

5 $$P_{g} = e^{{\left( {25.317 - \frac{5144}{{T_{g} + 273}}} \right)}}$$

*b)Radiation heat transfer between the glass cover and water* ($$Q_{r,wg}$$)*:* The radiation heat transfer between the glass cover and the water $$\left( {Q_{r,wg} } \right)$$ can be expressed in terms of an equivalent radiative heat transfer coefficient ($$h_{r} )$$ as in^30^ as follows:

6 $$Q_{r,wg} = h_{r,wg} A_{w} \left( {T_{w} - T_{g} } \right)$$

The equivalent radiative heat transfer coefficient between the glass cover and water can be obtained from the Stafan Boltzmann relation as follows^35^:

7 $$h_{r,wg} = \varepsilon_{effective} \sigma \left( {\left( {T_{w} + 273.15} \right)^{2} + \left( {T_{g} + 273.15} \right)^{2} } \right)\left( {T_{w} + T_{g} + 546.3} \right)$$

where $\sigma$ is Stefane Boltzmann constant, $A_{w}$ is the surface area of the water basin and $\varepsilon_{effective}$ is the effective emittance between the glass cover and the water surface that is given by:

8 $$\varepsilon_{effective} = \frac{1}{{\left[ {\frac{1}{{\varepsilon_{w} }} + \frac{1}{{\varepsilon_{g} }} - 1} \right]}}$$

*c)Evaporative Heat transfer between water and glass* ($Q_{e,wg}$)*:* Within the solar still, water evaporates due to heat transfer, rising as water vapor. This vapor condenses on the glass cover, releasing heat in the process. The evaporative heat transfer between the water surface and the glass cover represents the heat loss associated with this phase change which can be expressed as in^30,36^:

9 $$Q_{e,wg} = h_{e,wg} A_{w} \left( {T_{w} - T_{g} } \right)$$

The evaporative heat transfer between saline water and the glass is related to the convective heat transfer coefficient between the water and glass, $h_{c,wg} ,$ by the following relation as given by Velmurugan et al.^37^ and El Samanody et al.^38^:

10 $$Q_{e,wg} = 0.016237h_{c,wg } A_{w} \left( {P_{w} - P_{g} } \right)$$

From Eqns. (9) and (10), the evaporative heat transfer coefficient from water to the glass cover can be estimated as proposed by Yadav et al.^39^:

11 $$h_{e,wg} = 0.016237h_{c,wg} \left[ {\frac{{P_{w} - P_{g} }}{{T_{w} - T_{g} }}} \right]$$

#### Heat transfer outside the solar still

*a)Convective heat transfer between glass and ambient* ($Q_{c,ga}$)*:* Heat loss to the environment from the solar still primarily occurs through convection and radiation. Convective heat transfer plays a significant role, especially in the removal of heat from the glass surface and sidewalls of the solar still. As heat is transferred from the glass to the surrounding air through convection, the glass temperature decreases. This reduction in glass temperature enhances the temperature gradient between the water and glass, thereby intensifying the convective heat transfer process within the still. The efficiency of this heat removal process is influenced by factors such as wind speed and ambient temperature. Convective heat transfer between glass and ambient can be expressed as:

12 $$Q_{c,ga} = h_{c,ga} A_{g} \left( {T_{g} - T_{a} } \right)$$

where, the convective heat transfer coefficient, $h_{c,ga}$, can be represented as in terms of the wind movement, as in^38^:

13 $$h_{c,ga} = \left\{ {\begin{array}{*{20}c} \\ {2.8 + 3.0 W_{s} , for W_{s} \le 5m/s} \\ { 5.7 + 2.8 W_{s} , for W_{s} \ge 5 m/s} \\ \end{array} } \right.$$

*b)Radiative heat transfer between glass and ambient* ($Q_{r,ga}$)*:* Outside the solar still, the radiative heat transfer depends on the temperature of the glass, $T_{g}$, and sky temperature, $T_{sky} .$ Radiative heat transfer rate is given by Shukla et al.^40^ as follows:

14 $$Q_{r,ga} = \varepsilon_{g} \sigma A_{g} \left( {(T_{g} + 273.15)^{4} - (T_{sky} + 273.15)^{4} } \right)$$

In terms of the heat transfer coefficient $h_{r,ga}$, it can be expressed as:

15 $$Q_{r,ga} = h_{r,ga} A_{g} \left( {T_{g} - T_{a} } \right)$$

Hence, $h_{r,ga}$ can be expressed as:

16 $$h_{r,ga} = \varepsilon_{g} \sigma \left[ {\frac{{(T_{g} + 273.15)^{4} - (T_{sky} + 273.15)^{4} }}{{T_{g} - T_{a} }}} \right]$$

$T_{sky}$ is related to the ambient temperature ($T_{a}$) by the following formula as given by Duffie et al.^41^:

17 $$T_{sky} = 0.05525\left( {T_{a} + 273.15} \right)^{1.5} - 273.15$$

#### Solar still energy balance and calculations

*a) Energy balance of the glass surface:* The energy balance on the glass cover of the solar still involves several key components. Heat energy is lost from the outer surface of the glass to the environment through radiation and convection. Simultaneously, heat energy is released by the condensing water vapor inside the still, which is partially absorbed by the glass cover. Additionally, the energy balance accounts for the rate of energy change within the glass cover itself. Together, these factors determine the temperature of the glass cover ($T_{{gi}}$), as calculated by the following set of equations as per Taamneh et al.^42^ and Sathyamurthy et al.^43^ calculations;

18 $$\alpha _{g} I = h_{{to}} \left( {T_{{gi}} - T_{a} } \right) - h_{{ti}} \left( {T_{w} - T_{{gi}} } \right) + \frac{{m_{g} Cp_{g} }}{{A_{g} }}\frac{{dT_{g} }}{{dt}}$$

19 $$h_{to} = h_{c,ga} + h_{r,ga}$$

20 $$h_{ti} = h_{c,wg} + h_{e,wg} + h_{r,wg}$$

where $\alpha_{g}$ is glass absorptivity, $I$ is solar intensity, $h_{to}$ is the combined radiation and convection heat transfer coefficient between the outside glass surface and atmosphere and $h_{ti}$ is the total inner heat transfer coefficient between the water surface and glass cover of the solar still.

The time derivative in Eq. (18), using finite differencing, can provide the most recent temperature of the glass, $T_{{gi}}$, in terms of the temperatures of the previous time interval, $T_{{gi - 1}}$, where $i$ is the $i^{th}$ time step.

21 $$T_{gi} = \frac{{\alpha_{g} I + \left( {T_{w} h_{ti} } \right) + \left( {T_{a} h_{to} } \right) + \frac{{m_{g} C_{pg} }}{{A_{g} \Delta t}}T_{gi - 1} }}{{h_{ti} + h_{to} + \frac{{m_{g} C_{pg} }}{{A_{g} \Delta t}}}}$$

*b) Energy balance on the basin plate:* Convective heat transfer between the basin and the water and heat lost to the atmosphere through the sides make up the total amount of heat energy absorbed by the basin surface, taking into account the rate of energy change of the basin.

22 $$I\tau_{g} \tau_{w} \alpha_{b} A_{b} = Q_{c,bw} + Q_{c,ba} + I\tau_{g} \tau_{w} \tau_{b} A_{b} + m_{b} Cp_{b} \frac{{dT_{b} }}{dt}$$

where $\tau_{g}$ is glass transmittance, $\tau_{w}$ is water transmittance, $\tau_{b}$ is basin transmittance, $\alpha_{b}$ is basin absorptivity, $A_{b}$ is basin area, $Q_{c,ba}$ is the heat lost to the ambient.

The heat transfer due to convection between the basin and water ($Q_{c,bw}$) is expressed by Velmurugan et al.^37^ as:

23 $$Q_{{c,bw}} = h_{{c,bw}} A_{b} \left( {T_{{bi}} - T_{{wi}} } \right)$$

where $T_{{bi}}$ is basin temperature at time *i*, $T_{w}$ is basin water temperature at time *i*, $\alpha_{b}$ is basin absorptivity, $A_{b}$ is basin area, $h_{c,bw}$ is the heat transfer coefficient due to free convection between the horizontal plate (basin) and saline water and is expressed by Fujii et al.^44^ and Holman^45^ as:

24 $$h_{c,bw} = \frac{{0.15 k_{w} }}{L}\left( {Gr Pr} \right)^{\frac{1}{3}}$$

where $k_{w}$ is thermal conductivity of water, $L$ is the characteristic dimension which can be taken as the length of a side for a square or the mean of the two dimensions for a rectangular surface, $Gr$ is the average Grashof number and $Pr$ is the Prandtl number.

The heat lost to the surrounding ($Q_{c,ba}$) through the sides by convection is expressed in^30^ as follows:

25 $$Q_{{c,ba}} = h_{{ba}} A_{{side\;b}} \left( {T_{{bi}} - T_{a} } \right) = \left( {\frac{1}{{h_{{air}} }} + \frac{{d_{{ins}} }}{{k_{{ins}} }}} \right)^{{ - 1}} A_{{side\;b}} \left( {T_{{bi}} - T_{a} } \right)$$

where $h_{ba}$ is the overall heat transfer coefficient from basin to air, $k_{ins}$ is thermal conductivity of insulation, $A_{{sideb}}$ is basin plate side area, $d_{ins}$ is the insulation thickness, $T_{ins}$ is insulation surface temperature and $h_{air}$ is the convective heat transfer coefficient of air due to free convection between the vertical surface (insulation surface) and surrounding air and is expressed by Holman^45^ as

26 $$h_{air} = \frac{{ k_{air} }}{L}\left[ {0.825 + \frac{{0.387\left( {Gr Pr} \right)^{\frac{1}{6}} }}{{\left[ {1 + \left( {\frac{0.492 }{{Pr}}} \right)^{\frac{9}{16}} } \right]^{8/27} }}} \right]^{2}$$

From the above equations, the calculated basin temperature $T_{{bi}}$ at time *i*, in terms of the temperature of the previous interval, $T_{{bi - 1}}$, is written as

27 $$T_{{bi}} = \frac{{I\tau _{g} \tau _{w} \alpha _{b} - I\tau _{g} \tau _{w} \tau _{b} - h_{{ba}} \frac{{A_{{sideb}} }}{{A_{b} }}~T_{a} + h_{{cbw}} T_{{wi}} + \frac{{m_{b} C_{{pb}} }}{{A_{b} ~\Delta t}}T_{{bi - 1}} }}{{h_{{cbw}} + h_{{ba}} \frac{{A_{{sideb}} }}{{A_{b} }} + \frac{{m_{b} C_{{pb}} }}{{A_{b} ~\Delta t}}}}$$

*c) Energy balance on the basin water:* The energy balance of the basin water temperature is described as

28 $$I\tau _{g} A_{b} \alpha _{w} + Q_{{c,bw}} = m_{w} C_{{pw}} \frac{{dT_{w} }}{{dt}} + I\tau _{g} \tau _{w} A_{b} + h_{{ti}} A_{b} \left( {T_{{wi}} - T_{{gi}} } \right)$$

where $\alpha_{w}$ is water absorptivity, $\tau_{g}$ is glass transmittance, $A_{b}$ is basin area, $m_{w}$ is mass of basin water, $C_{pw}$ is water specific heat.

From Eq. (28) the outlet water temperature is determined at time *i*, in terms of the temperature of the previous interval, $T_{{wi - 1}}$, as

29 $$T_{wi} = \frac{{I\tau_{g} \alpha_{w} - I\tau_{g} \tau_{w} + h_{cbw} T_{bi} + h_{ti} T_{gi} + \frac{{m_{w} C_{pw} }}{{A_{w} \Delta t}}T_{wi - 1} }}{{h_{ti} + h_{cbw} + \frac{{m_{w} C_{pw} }}{{A_{w} \Delta t}}}}$$

*d) Mass of distillate produced of solar still:* The amount of water condensed on the inside surface of the glass represents the yield of the solar still and can be expressed as follows^39^:

30 $$M_{s} = 3600\left( {\frac{{Q_{e,wg} }}{{h_{fg} }}} \right)$$

where $h_{fg}$ is latent heat of water vaporization (J/kg).

#### Solar still section numerical calculation methodology

Using the initial temperature values of basin, water, and glass components of the solar still along with the ambient conditions, heat transfer energy and total heat transfer coefficients inside and outside the solar still may be obtained. These values are provided as input in the energy balance equations across basin, glass, and water in solar still assembly where the temperature values can be deduced along with the solar still flux productivity at time (*i*). The basin temperature is to be used as an input to the MD section thermal calculations as illustrated in the following section.

## Passive membrane performance model

In this section, the methodology will be presented to calculate the MD section distillate flux after merging it with the solar still. The passive membrane distillation uses the absorptivity properties of the hot wick (evaporator wick) which absorbs the brine from the solar still basin as previously shown in Fig. 1 while using the heat absorbed from solar basin base as thermal drive for the membrane distillation process to occur. Afterwards, the vapor passing through the membrane is condensed with a relatively colder wick (condenser wick) which transfers the distillate condensate to a separate collector.

### MD flux calculations

In this part, the distillate productivity as well as the thermal distribution across the MD stages will be calculated based on vapor pressure gradient, permeability, and thermal energy balance equations. For simplification, few factors have been considered in the following calculations:

1. The ternary mixture, consisting of water vapor, nitrogen, and oxygen, may be regarded as an ideal binary mixture of water vapor and air. Owing to the limited solubility of air in water, the air molecules confined within the pores remain essentially motionless.
2. Since there is no complete pressure differential across the membrane (unlike normal membrane distillation systems), the viscous flow term as well as the convective effects across the layers can be safely disregarded.
3. The brine used in the investigation has ion composition of sodium chloride content only.
4. The brine flow into the MD stages is constant.
5. The lateral convective heat loss per layer to atmosphere is negligible as the layer thickness is too small compared to other dimensions.
6. Condensed distillate has no salt content.

### Vapor pressure gradient on MD stages $\Delta p_{v}$

The N-stage distillation process operates based on the difference in water vapor pressure between the evaporating and condensing hydrophilic layers, which will be denoted by subscripts E and C in the following formulations, respectively. This pressure difference arises due to the variations in salinity and temperature through the air gap or hydrophobic membrane^46,47^. The resulting vapor pressure gradient can be estimated using Raoult’s law, as follows:

31 $$\Delta P_{v} = a_{E} \left( {Y_{E} } \right)P_{vE} \left( {T_{E} } \right) - a_{C} \left( {Y_{C} } \right)P_{vC} \left( {T_{C} } \right)$$

where $a_{E}$ and $a_{C}$ denote the activity of water of evaporator and condenser sides respectively, $Y_{E}$ and $Y_{C}$ are the mass fractions of salt in the feed and distilled solution, respectively, $P_{v}$ is the water vapour pressure, and $T_{E}$ and $T_{C}$ are the temperatures of the saline and distillate solutions, respectively^48^, which will be calculated in the following section. Under ideal conditions, the activity of an aqueous NaCl solution can be estimated as:

32 $$a_{E} = \frac{{M_{{NaCl}} \left( {1 - Y_{E} } \right)}}{{M_{{NaCl}} \left( {1 - Y_{E} } \right) + N_{{ionE}} M_{{H_{2} O}} Y_{E} }}$$

where $M_{NaCl}$ and $M_{{H_{2} O}}$ are the molar weights in gram per mole of sodium chloride (58.44 g/mol) and water (18.02 g/mol), respectively, and $N_{ion}$ = 2 for sodium chloride. Because the feed water used in the tests has a salinity of 35 g/l ($Y_{E}$ = 0.035), which is normal for seawater, Eq. (32) shows that activity of the saline water is 0.98 and the activity of the distilled water is 1. The Antoine semi-empirical correlation can be used to evaluate the vapor pressure^49^:

33 $$\log \left[ {P_{v} } \right] = A - \frac{B}{C + T}$$

where $P_{v}$ is intended in mmHg and $T$ in Celsius, and A, B and C are material specific constants, in this case equal to 8.07, 1730.63 and 233.42, respectively.

### Permeability equation *K*

The specific mass flow rate of the distillate $J$, (${\text{kg}}/{\text{m}}^{2} {\text{s}}$) can be determined by combining the Maxwell–Stefan and dusty-gas models^50–52^. Both molecular diffusion and the external driving force, which includes the concentration effect or chemical potential, are included in the Maxwell–Stefan model (which describes the interaction between gas molecules).

Elimelech and colleagues^50–52^ have extensively discussed a series of assumptions that lead to the following equation:

34 $$J = K\Delta P_{v}$$

where $\Delta P_{v}$ is derived from Eq. (31).

In this equation, $J$ is linearly dependent on the partial pressure gradient via the permeability coefficient ($K$) of the space between the two hydrophilic layers^29^.

When transition flow dominates in each stage of the distiller, the overall empirical permeability coefficient, as reported in Eq. (34), can be approximated as the sum of contributions from the membrane and spacer permeability^26,46^. This relationship is expressed as:

35 $$\frac{1}{K} = \frac{1}{{\frac{{M_{{H_{2} O}} PD_{{wa}} \epsilon _{m} }}{{p_{{air}} RT\tau d_{m} }}}} + \frac{1}{{\frac{{2M_{{H_{2} O}} \epsilon _{m} r}}{{3RT\tau d_{m} }}\sqrt {\frac{{8RT}}{{\pi M_{{H_{2} O}} }}} }} + \frac{1}{{\frac{{M_{{H_{2} O}} PD_{{wa}} \epsilon _{s} }}{{p_{{air}} RTd_{{air}} }}}}$$

The equation provided represents the overall mass transfer coefficient $K$ in a system involving water vapor and air. This coefficient quantifies the efficiency of mass transfer across a boundary, considering various resistances. The first term in the equation accounts for the resistance due to diffusion through the medium, where $M_{{H_{2} O}}$ is the molar mass of water, $P$ is the total pressure, $D_{wa}$ is the diffusion coefficient of water vapor in air which is empirically estimated as follows^53^:

36 $$PD_{wa} = 1.19{*}10^{ - 4} T^{1.75}$$

$\epsilon_{m}$ is the porosity of the medium, $p_{air}$ is the partial pressure of air, $R$ is the universal gas constant, $T$ is the absolute temperature, and $d_{m}$ is the thickness of the medium. The second term represents Knudsen diffusion, significant when the pore radius $r$ is comparable to the mean free path of the molecules, incorporating the kinetic theory of gases. The third term addresses the resistance due to diffusion through an air layer, where$\epsilon_{s}$ is the surface porosity and $d_{air}$ is the thickness of the air layer. Each term plays a crucial role in determining the overall mass transfer rate, with temperature $T$ being particularly influential as it affects molecular motion and diffusion coefficients. Understanding these terms helps in analyzing and optimizing mass transfer processes, especially under varying temperature gradients.

It is noteworthy that the linearized model in Eq. (35) has been successfully applied in numerous membrane distillation experiments reported in the literature^54,55^. It yields predictions that closely align with both the Maxwell–Stefan and dusty-gas models^29^.

### Passive membrane energy balance

**Fig. 4:**
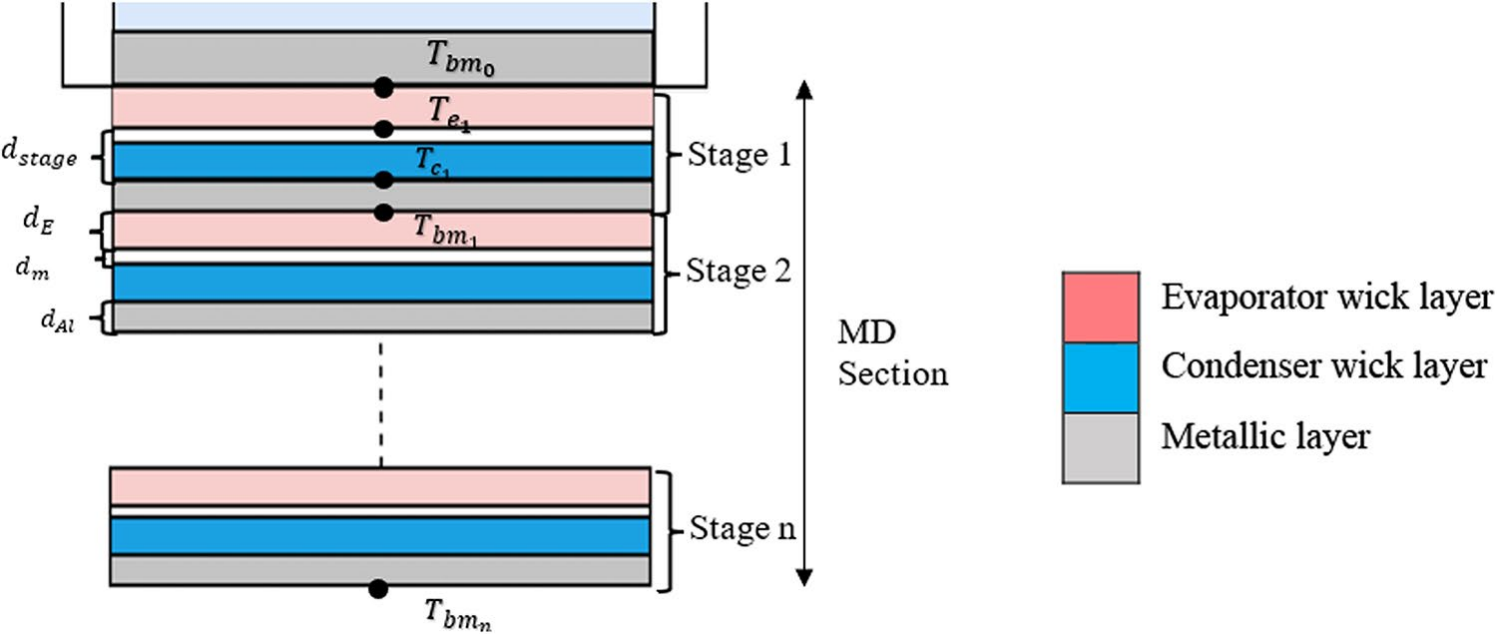
Temperature distribution along MD for n stages used in the parametric study.

The overall gap thickness of each distiller stage which includes the air gap $d_{a}$, and/or membrane thicknesses $d_{m}$, has a significant impact on the permeate flow. In this case, DCMD approach is being investigated which indicates the absence of air gap between the stage layers as shown in Fig. 4.

It is important to note that the water flows at the entrance (saltwater) and exit (distillate) of the stagnant wicks are entirely driven by capillarity, resulting in very low Reynolds numbers^29^. In the feed and permeate channels (i.e., hydrophilic layers), convection is weak, and heat transfer is mostly governed by conduction. This contrasts with conventional membrane distillation methods (sweeping gas, air-gap, and direct contact MDs), which have problems with temperature polarization^29^.

To achieve high permeability, consequently, a high mass flow, it is essential to balance the restriction of heat transfer with the enhancement of mass movement between the layers^46,47^. The specific heat flow (${{q}}^{{\prime \prime }}$, W/m^2^) between the evaporating and condensing hydrophilic layers in each step of the distiller is primarily driven by water phase changes and heat transfer through conduction as follows:

37 $$Q = \frac{{k_{stage} }}{{d_{stage} }}\left( {T_{E} - T_{C} } \right) + K\Delta P_{v} \Delta h_{lv}$$

where, $T_{E}$ and $T_{C}$ are the temperatures of the feed and permeate solution, while $d_{stage}$ is the effective thickness value of this layer which is the total thickness of the membrane and condenser wick and $k_{stage}$ is the effective thermal conductivity in this layer per stage, considering conduction via membrane and is expressed as follows:

38 $$k_{stage} = \left( {\frac{1}{{k_{m} }} + \frac{1}{{k_{C} }}} \right)^{ - 1}$$

where $k_{m}$ and $k_{C}$ are the thermal conductivity values of membrane and condenser wicks, respectively.

**Fig. 5:**
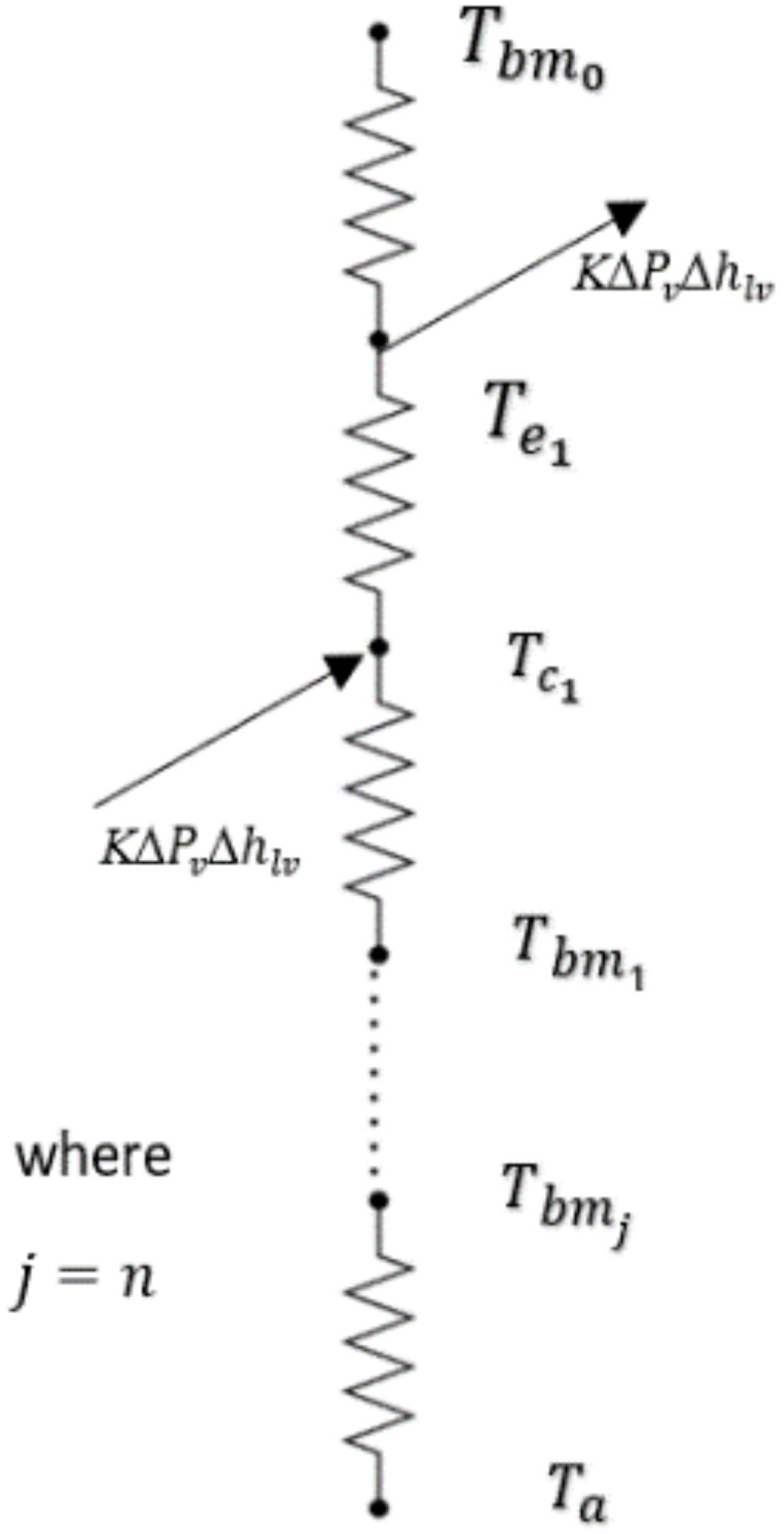
Thermal resistance diagram of the MD section.

As illustrated in Fig. 5, the remaining layers can be calculated using the energy balance equations by considering the thermal conductivity, layer thickness, and heat loss for each layer, while neglecting insulation and convective losses on the membrane distillation side. It is also important to note that the term *K∆*$P_{v}$*∆*$h_{lv}$ represents the heat lost due to phase change, either through evaporation or condensation, where *∆*$h_{lv}$ is the latent heat of vaporization^56^.

The passive membrane energy balance calculations will be done in order to deduce the temperature distribution along the MD section layers as well as to highlight the significance of the thermal drive from the solar energy applied to the bottom basin area which will be considered as the starting point in the energy balance calculations till reaching the bottom MD section base metal exposed to the atmosphere.

By applying the energy balance to each layer within the membrane distillation, a set of equations that provide three unknown temperature values for each layer: evaporator (*e*), condenser (*c*) base metal/bottom layer (*bm*) for each stage (*j*) across the total number of stages (n) can be obtained. These temperature values are determined by the following set of equations:

- Evaporator layer:
  39 $$\frac{{k_{E} \left( {T_{{bm_{j} }} - T_{ej} } \right)}}{{d_{E} }} = \frac{{k_{stage} (T_{ej} - T_{{}} )}}{{d_{stage} }} + K \Delta P_{v} \Delta h_{lv}$$
  where $T_{{bm_{j} }} = T_{bi}$ (calculated in Eq. (27)), if $j = 1$, where $j$ is the stage number.
- Condenser layer:
  40 $$\frac{{k_{stage} (T_{ej} - T_{cj} )}}{{d_{stage} }} + K\Delta P_{v} \Delta h_{lv} = \frac{{k_{Al} (T_{cj} - T_{bmj} )}}{{d_{Al} }}$$
- Base Metal layer:
  41 $$\frac{{k_{Al} (T_{cj} - T_{bmj} )}}{{d_{Al} }} = a\frac{{k_{E} (T_{bmj} - T_{ej + 1} )}}{{d_{E} }} + b \left[ {h_{air} \left( {T_{bmj} - T_{a} } \right)} \right]$$

The flags $a$ and $b$ are determined based on the order of the membrane layer as follows:

- for all layers except the last one ($$j < n) , a = 1, b = 0$$
- for the last layer $\left( {j = n} \right)$,$a = 0, b = 1$

Therefore, using the previous equations, the solution methodology of the software at time step (*i*) is considered to be a double loop iterative type of solution where the three unknowns per stage ( *j*) $T_{e}$, $T_{c}$, $T_{bm}$ are deduced by adding the basin temperature $T_{bi}$ as an input temperature with an output of ambient temperature $T_{a}$ by which the heat is transferred out of the assembly by means of convection, indicating double loop solution for time step (*i*) among all (*j*) stages.

## Results and discussions

### Parametric study

The model is incorporated into the Engineering Equation Solver (EES)^57^ software, allowing for the simultaneous solution of non-linear equation systems using the Newton–Raphson method. Proper initialization for parameters is crucial for the model to converge successfully. Additionally, the EES software includes libraries for the thermophysical characteristics of pure water and vapor based on the IAPWS Formulation 1995^58^ as well as the properties of saltwater consistent with the correlations provided by Sharqawy et al.^59^.

### 5.2 Mathematical model validation

**Fig. 6:**
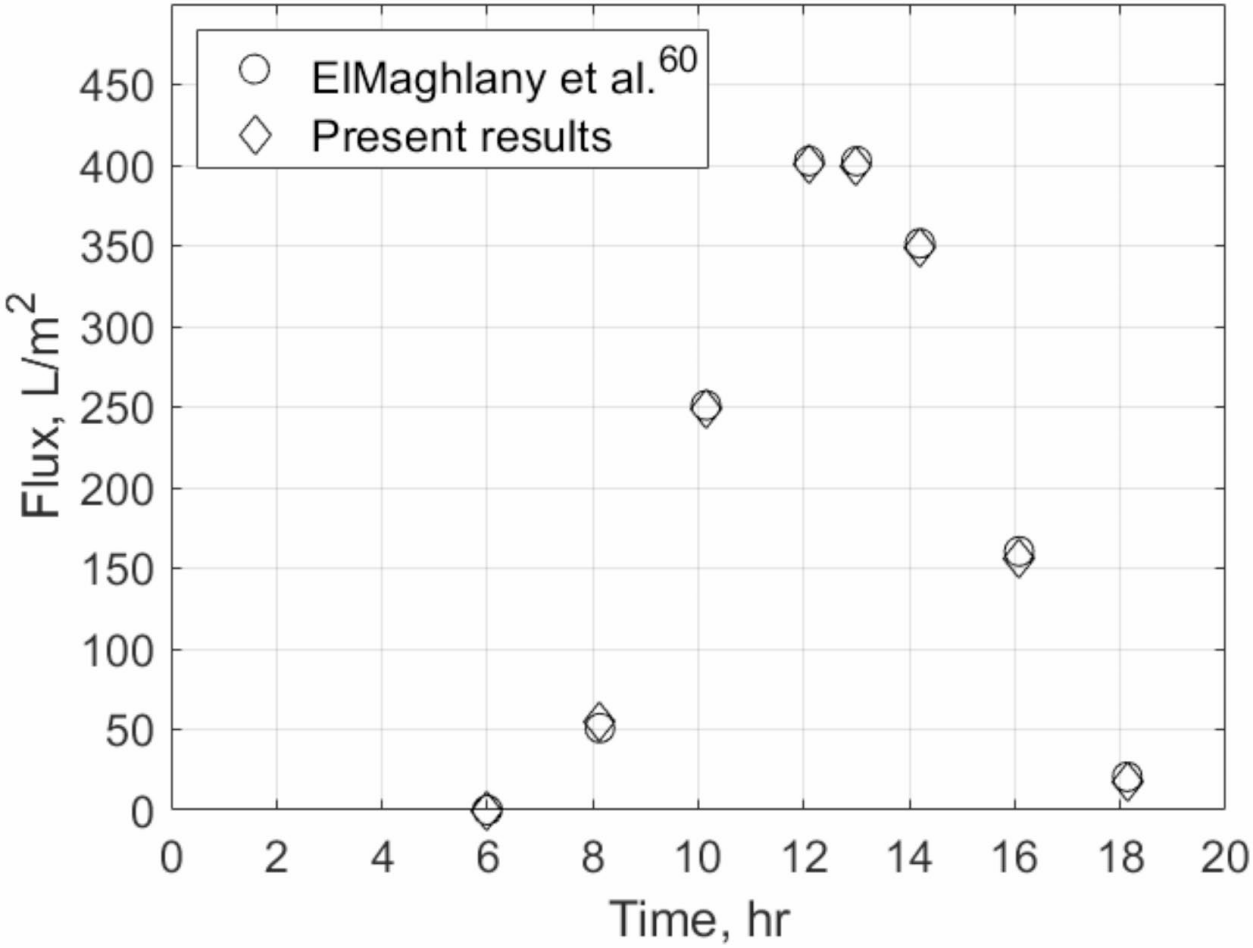
Validation with single basin solar still daily flux data of El-Maghlany et al. ^60^.

**Fig. 7:**
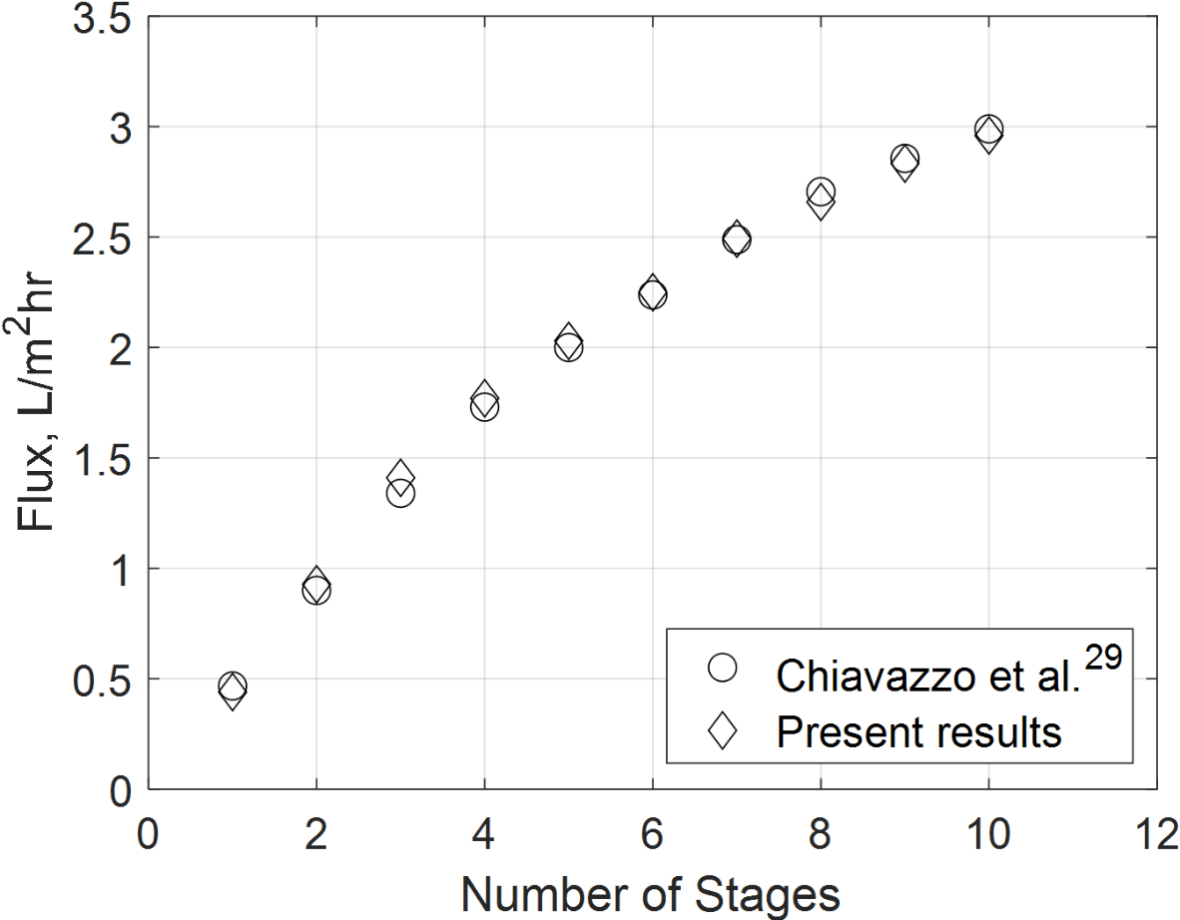
Validation with the membrane flux of Chiavazzo et al. results ^29^.

The model is validated using experimental data from two studies: one by ElMaghlany et al.^60^ for a conventional solar still and another by Chiavazzo et al.^29^ for a passive membrane distillation system. The validation process, illustrated in Figs. 6 and Fig. 7, is conducted in two steps.

For the conventional solar still, the model’s predictions showed strong agreement with the experimental data, with relative deviations below 5%. These minor discrepancies are attributed to factors such as variations in solar flux intensity, unaccounted thermal losses to the surroundings, experimental measurement uncertainties, and assumptions in the evaporation and condensation processes. The heat transfer balance equations (e.g., Eqs. (18), (22) and (24) from Section “Solar Still energy balance and calculations”) accurately describe the energy exchange within the system, ensuring reliable predictions of temperature and evaporation rates. This close alignment confirms the robustness of the model in simulating the solar still’s performance.

In the case of passive membrane distillation, the model also demonstrated excellent agreement with the experimental results, with deviations within 5%. These differences primarily arise from the assumptions and approximations in the membrane properties and derived parameters used in the model. Specifically, the membrane properties, such as pore size, porosity, and thickness, are derived from idealized conditions in the model, whereas the experimental setup may exhibit slight variations due to manufacturing tolerances or operational wear. For instance, the pore size distribution in the experimental membrane may not be perfectly uniform, leading to small deviations in mass transfer rates compared to the model predictions, which assume a uniform pore size.

Additionally, the derived parameters in the model, such as the heat and mass transfer coefficients, are based on theoretical correlations and may not fully capture the complex interactions occurring in the experimental system. For example, the heat transfer coefficient between the membrane and the surrounding air may vary slightly due to local airflow conditions or temperature gradients that are not explicitly accounted for in the model. Similarly, the mass transfer coefficient, which is critical for predicting flux in membrane distillation, is derived from theoretical relationships (e.g., Eq. (31) for vapor pressure and Eq. (35) for flux) and may not fully reflect the experimental conditions, such as minor fluctuations in temperature or humidity.

Despite these deviations, the close alignment between the model and experimental data confirms the robustness of the mathematical formulation. Equation (31), which governs the vapor pressure difference across the membrane, accurately captures the relationship between temperature and vapor pressure, demonstrating its reliability in predicting the driving force for mass transfer. Similarly, Eq. (35), which incorporates the vapor pressure difference and membrane properties, effectively predicts the flux across the membrane, aligning well with the experimental trends. These equations, along with the derived parameters, provide a strong foundation for the model, ensuring its accuracy and reliability.

The validation results underscore the model’s ability to simulate real-world scenarios with high precision, making it a valuable tool for further analysis and optimization. By accounting for the key parameters and membrane properties, the model offers a reliable framework for predicting system performance under varying conditions, while the minor deviations highlight areas for potential refinement in future studies.

### Base case

The model is computed for the base case using conventional solar still input data from Sathaumurthy et al.^43^ and passive membrane data from Chiavazzo et al.^29^ with a hydrophobic PTFE membrane featuring a pore size of 0.1 µm. The analysis considered several factors influencing the overall distillate productivity of the design. All derived parameters and empirical constants in the heat transfer balance equations aligned with the base case study. The survival probability of the integrated SS-MD system is evaluated based on its ability to maintain optimal performance under varying operational and environmental conditions. Key parameters analyzed include:

- *1. Solar flux:* Seasonal variations in solar intensity and daytime duration.
- *2.Brine depth:* Depth of brine in the SS basin, influencing thermal mass and evaporation rates.
- *3.Wind speed:* Affecting convective cooling and evaporation efficiency.
- *4. Number of MD stages:* Determining heat recovery and vapor transport efficiency.
- *5.Initial brine temperature:* Baseline condition for system performance evaluation.
- *6.Residual temperature:* Reflecting thermal efficiency and heat utilization.
- *7.Climatic conditions:* Seasonal variations impacting energy availability.

These parameters collectively determine the system’s ability to sustain high productivity and efficiency under diverse operating conditions, ensuring its long-term performance. The numerical testing methods for evaluating these parameters are summarized in Table 1.

**Table 1:** Summary of test parameters and types of numerical tests conducted on the integrated SS-MD system.

Parameters	Values/details	Type of test
SS section		
Solar flux	June, March, December	Seasonal performance analysis (effect of solar intensity and daytime duration)
Brine depth	0.5 mm, 1.0 mm, 1.5 mm	Sensitivity analysis (effect of brine depth on productivity)
Brine initial temperature	25°C	Fixed parameter (baseline condition)
Wind speed	2 m/s, 4 m/s, 6 m/s	Sensitivity analysis (effect of wind speed on convective cooling)
MD section		
Number of stages	Single-stage, two-stage, three-stage, …, ten-stage	Performance optimization (effect of number of stages on productivity)

### Effect of number of MD stages on the accumulated daily distillate flux

**Fig. 8:**
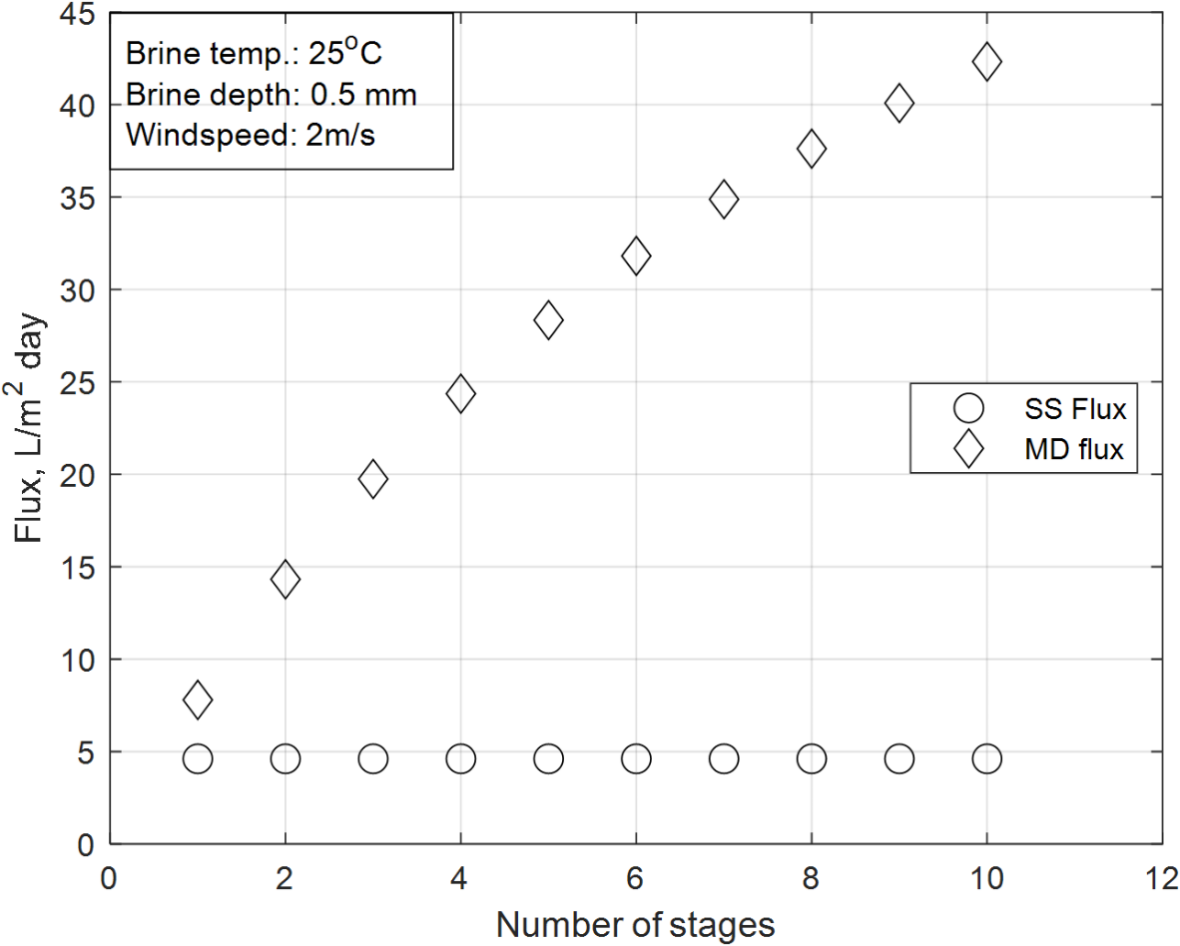
Detailed distillate productivity of both SS and MD sections for the same stage.

Figure 8 illustrates the accumulated productivity of the solar still (SS) and membrane distillation (MD) sections across varying numbers of MD stages under consistent climatic conditions (solar flux of June, wind velocity of 2 m/s, brine initial temperature of 25 °C, and brine depth of 0.5 mm). The SS section exhibits a constant productivity of 4.6 ${\text{L}}/{\text{m}}^{2} {\text{day}}$ across all trials, as its performance is governed by the heat transfer balance equations. The constant productivity of the SS section is attributed to the fixed solar flux and basin parameters, as described by this equation.

In contrast, the MD section shows a significant increase in productivity, starting at 7.8 ${\text{L}}/{\text{m}}^{2} {\text{day}}$ for a single-stage configuration and rising steadily to 42.3 ${\text{L}}/{\text{m}}^{2} {\text{day}}$ for a ten-stage configuration. This trend is explained by the principles of thermally localized membrane distillation, as proposed by Chiavazzo et al.^29^, and is directly related to Eq. (35), which describes the membrane permeability ($K$).

The increase in productivity with the number of MD stages is driven by the following factors, as captured by Eq. (35):

- *1.Enhanced temperature and pressure gradients:* As the number of stages increases, the temperature gradient across the system becomes more pronounced. This directly impacts the pressure gradient in Eqs. (31) and (35), which enhances vapor transport through the membrane. The term $\frac{1}{{p_{air} RT\tau d_{m} }}$ in Eq. (35) reflects the influence of pressure and temperature on permeability, explaining the higher productivity in multi-stage configurations.
- *2.Efficient heat recovery:* Each additional stage acts as a heat recovery unit, transferring latent heat from the vapor to the incoming feed water. This reduces thermal losses and increases the overall efficiency of the system, as reflected in the temperature-dependent terms in Eq. (35) (e.g., $\sqrt {\frac{8RT}{{\pi M_{{H_{2} O}} }}}$). The efficient heat recovery in multi-stage configurations ensures that more thermal energy is utilized for vapor transport, further boosting productivity.

The results demonstrate the dominance of the MD section in contributing to the overall distillate flux, accounting for most of the daily productivity compared to the SS section. The integrated system achieves an overall productivity approximately five times higher than that of a standalone SS system in the ten-stage configuration. This enhancement is directly attributed to the mechanisms described by Eq. (35), which governs membrane permeability and vapor transport, validating the efficacy of the proposed design.

### Effect of number of MD stages on the hourly distillate flux

**Fig. 9:**
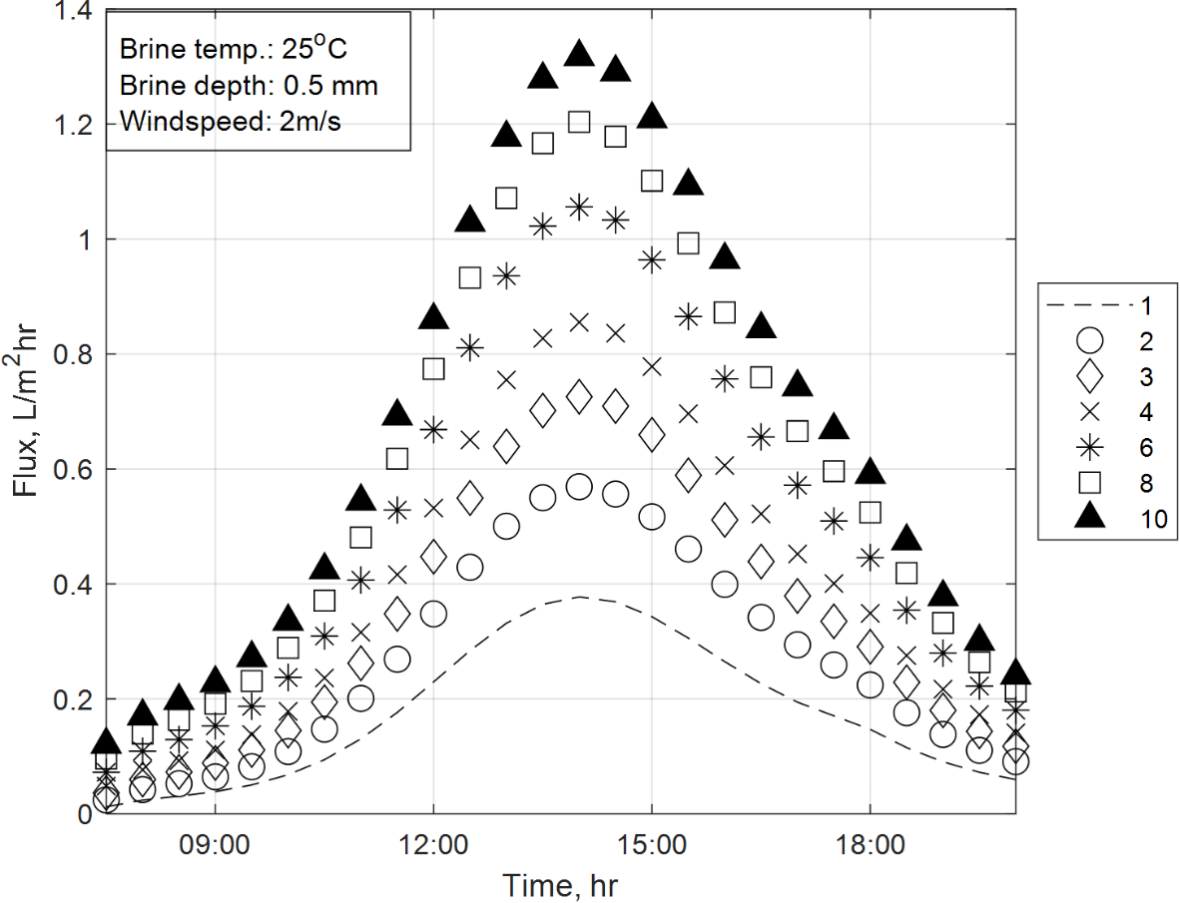
Hourly distillate production of the proposed novel design vs number of stages (n).

Figure 9 depicts the instantaneous hourly distillate productivity of the integrated SS-MD system during daytime (8:00 AM to 8:00 PM) under constant climatic conditions (solar flux of June, wind velocity of 2 m/s, brine initial temperature of 25 °C, and brine depth of 0.5 mm). The productivity is calculated at 10-min intervals, and the results are shown for different numbers of MD stages.

The figure indicates that the hourly distillate flux rate increases with the number of stages, reaching its maximum output in the ten-stage configuration. This trend is linked to the heat and mass transfer mechanisms described by Eq. (35) and the energy balance equations governing the system. Three major observations can be distinguished during this analysis, specifically:

1. Mid-day productivity surge:
  During mid-day (when solar flux is highest), the two-stage configuration shows the largest relative increase in productivity (50%) compared to the single-stage configuration. This surge is attributed to the efficient heat recovery in the two-stage system, which maximizes the utilization of solar energy for vapor transport, as described by Eq. (35).
  The temperature and pressure gradients across the membrane are highest during mid-day, enhancing vapor transport and resulting in a significant productivity boost.
2. Diminishing Returns with additional stages:
  As the number of stages increases beyond two, the relative increase in productivity diminishes. For example, the three-stage configuration shows only a 27% increase compared to the two-stage configuration, while the ten-stage configuration shows just an 8% increase compared to the eight-stage configuration.
  This trend is explained by the concept of diminishing returns in heat recovery. While additional stages improve productivity, the marginal gains decrease as the system approaches its maximum thermal efficiency. This behavior is captured by the temperature-dependent terms in Eq. (35), which shows that the incremental impact of additional stages on vapor transport becomes smaller as the number of stages increases.
3. Maximum output in ten-stage configuration:
  The ten-stage configuration achieves the highest hourly productivity, as it fully utilizes the available solar energy and maximizes heat recovery. However, the rate of increase in productivity slows down significantly beyond eight stages, highlighting the trade-off between system complexity and performance gains.

The results demonstrate that the number of MD stages has a significant impact on the system’s hourly productivity, particularly during peak solar hours. The trends observed in Fig. 8 are consistent with the mechanisms described by Eq. (35) and the energy balance equations, validating the efficacy of the integrated SS-MD design.

### Effect of increasing brine depth in SS basin section on the overall accumulated distillate flux

**Fig. 10:**
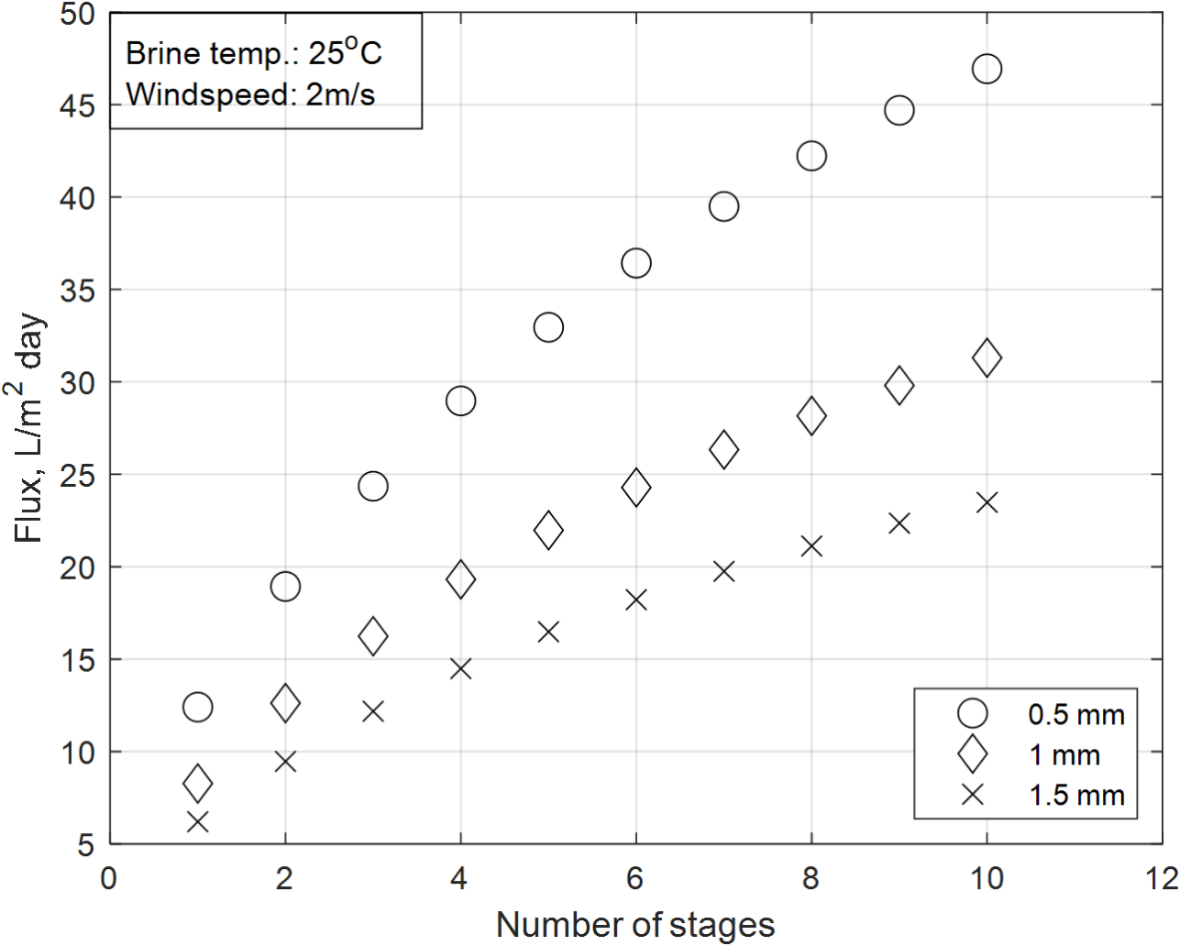
Effect of brine depth on the overall accumulated distillate flux productivity.

Figure 10 shows the effect of increasing the brine depth in the SS basin on the overall accumulated distillate productivity. The investigation is conducted under constant climatic conditions (solar flux of June, wind velocity of 2 m/s, and initial brine temperature of 25 °C), with brine depths varied in 0.5 mm intervals. The results indicate that a brine depth of 0.5 mm yields the highest productivity across all ten MD stage configurations, while a depth of 1.5 mm results in the lowest productivity. Additionally, the difference in productivity between the 0.5 mm trials and other depths increases progressively from the single-stage configuration to the ten-stage configuration.

This trend is explained by the physics of heat transfer and evaporation in the SS section:

1. Impact of brine depth on productivity:
  The brine depth directly influences the thermal mass of the water in the SS basin. A thin brine layer (0.5 mm) has a lower thermal mass, allowing the water to heat up more quickly under the same solar intensity. This rapid temperature rise maximizes the energy available for evaporation, leading to higher distillate productivity. The thin layer also reduces thermal inertia, ensuring that more of the absorbed solar energy is used for vapor generation rather than heating the water.
  In contrast, a thicker brine layer (1.5 mm) increases the thermal mass, requiring more energy to raise the water temperature. This results in a slower temperature rise and lower evaporation rates, as more energy is lost to the surroundings rather than being used for vapor generation. Consequently, productivity decreases with increasing brine depth.
2. Productivity change with number of stages:
  The difference in productivity between the 0.5 mm trials and other depths becomes more pronounced as the number of MD stages increases. This is because the temperature gradient across the system is more sensitive to brine depth in multi-stage configurations.
  In the ten-stage configuration, the cumulative effect of heat recovery and vapor transport amplifies the impact of brine depth on productivity. The thin brine layer in the 0.5 mm trials ensures that more thermal energy is transferred to the MD section, enhancing vapor transport and flux productivity as described by Eq. (35).

The results demonstrate that brine depth is a critical parameter influencing the performance of the integrated SS-MD system. Lower brine depths maximize productivity by optimizing the energy available for evaporation, while higher brine depths reduce productivity due to increased thermal losses and slower temperature rise. These findings are consistent with the mechanisms described by the energy balance and permeability equations, validating the importance of optimizing brine depth in the design of solar desalination systems.

### Effect of climatic conditions on the overall accumulated distillate flux

**Fig. 11:**
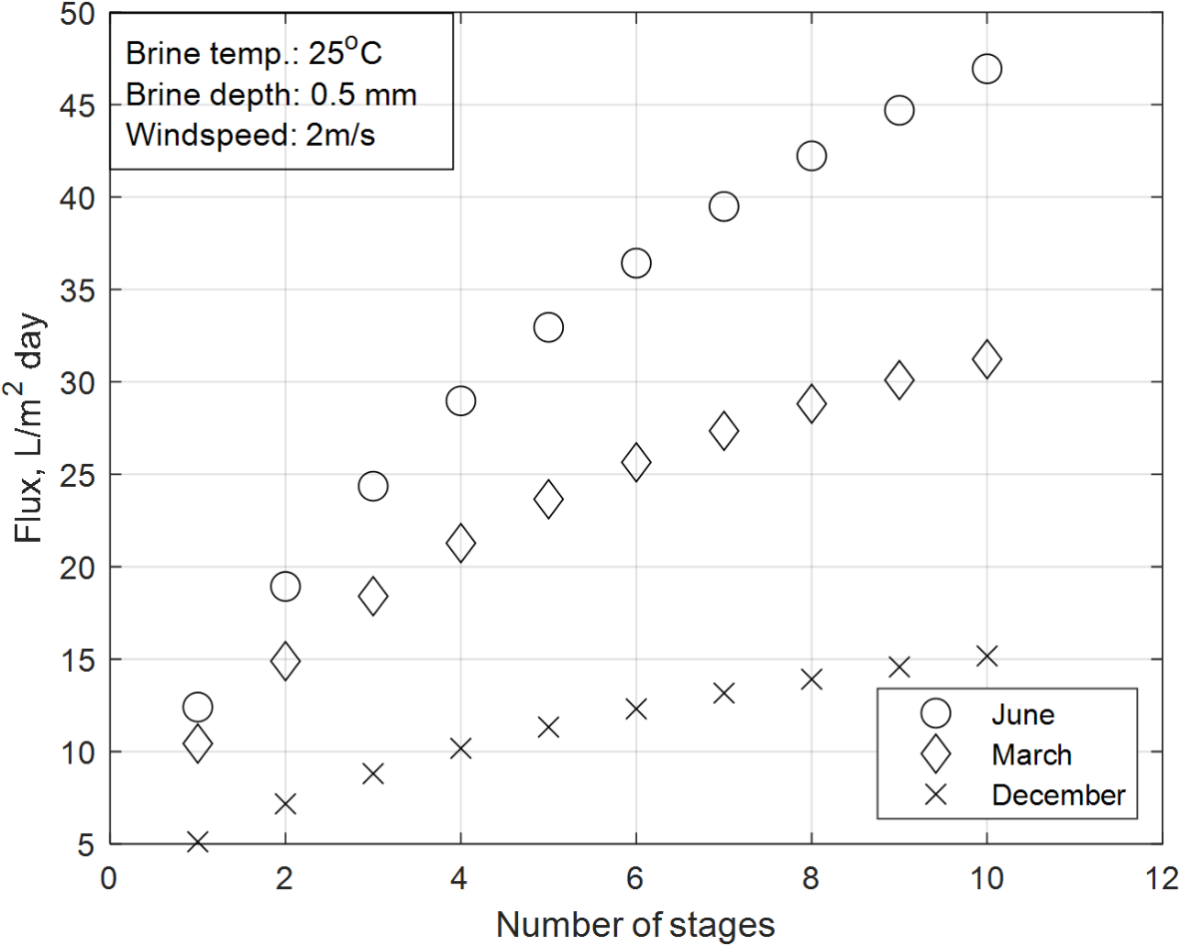
Effect of different seasonal climatic parameter on the overall accumulated distillate flux.

Figure 11 reveals the accumulated daytime distillate flux for the integrated SS-MD system under different seasonal conditions (June, March, and December). The investigation is conducted with constant parameters (wind velocity of 2 m/s, brine initial temperature of 25 °C, and brine depth of 0.5 mm) across all trials. The results show that the accumulated productivity is highest in June, reaching 46.94 ${\text{L}}/{\text{m}}^{2} \;{\text{day}}$ in the ten-stage configuration, followed by 31.23 ${\text{L}}/{\text{m}}^{2} \;{\text{day}}$ in March and 15.18 ${\text{L}}/{\text{m}}^{2} \;{\text{day}}$ in December. Additionally, the impact of the number of MD stages on productivity is more pronounced in June compared to other seasons, as the rate of decrease in productivity with increasing stages is lowest in June and highest in December.

These trends are explained by the seasonal variations in solar intensity and daytime duration, which directly influence the system’s performance:

1. Seasonal impact on productivity:
  June has the highest solar intensity and the longest daytime duration among the three seasons. This results in a greater amount of sensible heat absorption in the SS section, which increases the evaporation rate and provides more thermal energy for the MD section. The higher solar flux in June ensures that the temperature and pressure gradients across the system are maximized, enhancing vapor transport through the membrane as described by the permeability equation. This leads to higher productivity, particularly in multi-stage configurations.
  In contrast, March and December have lower solar intensity and shorter daytime durations, resulting in less sensible heat absorption and lower evaporation rates in the SS section. Consequently, less thermal energy is available for the MD section, leading to reduced productivity. The lower solar flux in these seasons also reduces the temperature and pressure gradients across the system, limiting vapor transport through the membrane as described by the permeability equation.
2. Impact of Number of Stages:
  The number of MD stages has a greater impact on productivity in June compared to other seasons. This is because the higher solar intensity in June provides sufficient thermal energy to support heat propagation across multiple stages, maximizing the benefits of heat recovery and vapor transport.
  In contrast, the lower solar intensity in March and December limits the system’s ability to utilize additional stages effectively, resulting in smaller productivity gains with increasing stages.

The results demonstrate that solar intensity and daytime duration are critical factors influencing the performance of the integrated SS-MD system. Higher solar intensity and longer daytime durations, as seen in June, maximize productivity by providing more thermal energy for evaporation and vapor transport. These findings are consistent with the mechanisms described by the permeability equation and the energy balance principles, validating the importance of considering seasonal variations in the design and operation of solar desalination systems.

### Effect of MD stages on the average bottom residual temperature

**Fig. 12:**
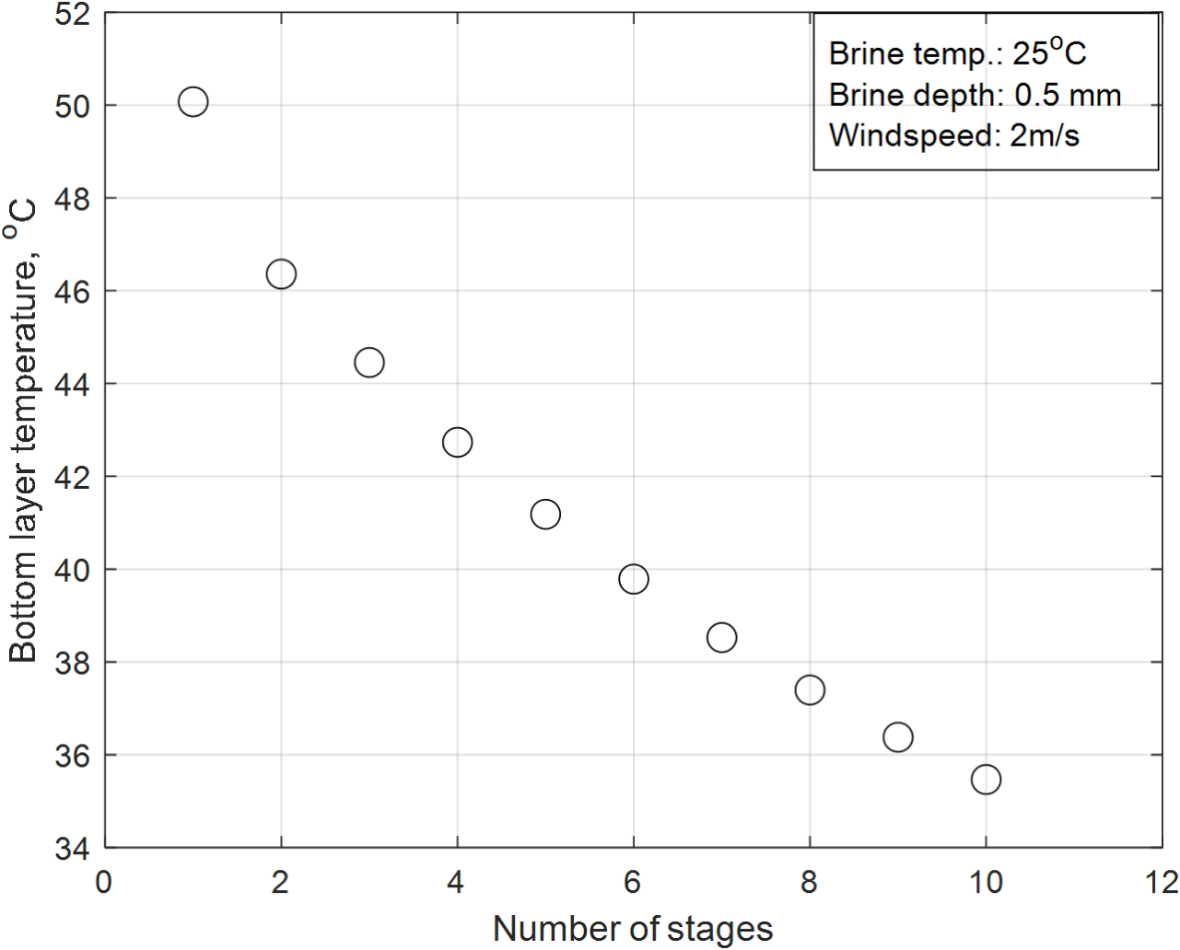
Average residual temperature values for each stage.

Figure 12 depicts the bottom residual average temperature of the MD section for the proposed design across different numbers of stages. The investigation is conducted under constant climatic conditions (solar flux of June, wind velocity of 2 m/s, brine initial temperature of 25 °C, and brine depth of 0.5 mm). The results show that the single-stage MD configuration has the highest residual temperature (50.8 °C), which decreases as the number of stages increases, reaching a minimum of 35.5 °C in the ten-stage configuration—close to the ambient temperature in June. These trends are explained by the heat transfer and temperature gradient dynamics in the MD section, as described by Eqns. 39–41, which govern the temperature distribution across the evaporator, condenser, and base metal layers. Specifically:

1. Impact of number of stages on residual temperature:
  In the single-stage configuration, the temperature gradient between the hot and cold sections of the MD stage is maximized. This results in a higher residual temperature at the bottom of the MD section, as more thermal energy is retained in the system. However, the high residual temperature also indicates significant thermal energy losses, as a substantial portion of the absorbed solar energy is not effectively utilized for vapor transport. This reduces the overall thermal efficiency of the system.
  As the number of stages increases, the temperature gradient across each stage becomes smaller. This is because the thermal energy is distributed more evenly across the system, reducing the residual temperature at the bottom of the MD section. The lower residual temperature in multi-stage configurations (e.g., 35.5 °C in the ten-stage configuration) indicates that more thermal energy is being utilized for vapor transport, improving the overall efficiency of the system. This is consistent with the principles of heat recovery and thermal localization in membrane distillation, as described by Eqns. 39–41.
2. Effect of temperature gradient:
  The temperature gradient between the hot and cold sections of each MD stage is the primary driver of vapor transport as described in Eq. (31). A higher gradient enhances vapor flux, while a lower gradient reduces it. The results in Fig. 12 highlight the trade-off between residual temperature and thermal efficiency, emphasizing the importance of optimizing the number of stages to balance these factors.

The results demonstrate that the number of MD stages significantly impacts the residual temperature and thermal efficiency of the system. While single-stage configurations retain more thermal energy, they suffer from higher losses and lower efficiency. In contrast, multi-stage configurations distribute thermal energy more effectively, reducing losses and improving efficiency. These findings are consistent with the heat transfer principles and the permeability equation, validating the importance of optimizing the number of stages in the design of solar desalination systems.

### Wind speed effect on the total daily distillate flux:

**Fig. 13:**
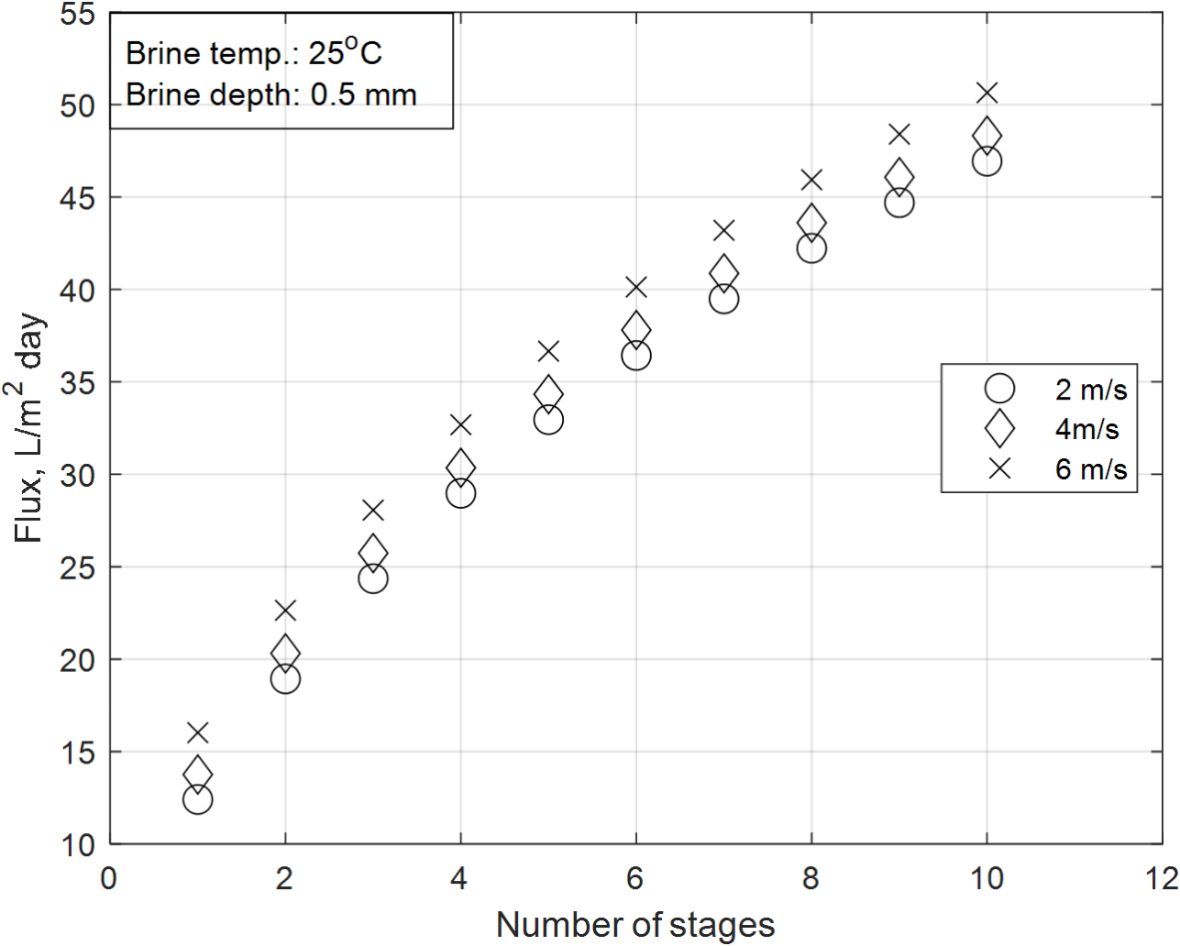
Wind speed impact on overall daily distillate flux.

Figure 13 shows the impact of wind speed on the total daily distillate flux of the integrated SS-MD system. The investigation is conducted under constant climatic conditions (solar flux of June, brine initial temperature of 25 °C, and brine depth of 0.5 mm), with wind speeds varied from 2 to 6 m/s. The results indicate that increasing the wind speed by an average of 2 m/s leads to an increase in the total daily flux by approximately 3% (from 2 to 4 m/s) and 5% (from 4 to 6 m/s). Additionally, the performance profile of the system remains consistent across all wind speed trials, with similar rates of increase in productivity.

These trends are explained by the physics of convective heat transfer in the SS section, as described by Eq. (12), and its impact on the overall system performance can be recognized in the following highlights:

1. Impact of wind speed on SS section:
  The SS section is sensitive to wind speed due to its reliance on convective heat transfer at the water surface. Higher wind speeds enhance the convective cooling of the SS basin, increasing the temperature gradient between the water surface and the surrounding air. This results in higher evaporation rates and, consequently, higher distillate productivity.
  The increase in productivity with wind speed is consistent with the heat transfer principles governing the SS section, as described by Eq. (12). This equation highlights the relationship between wind speed, convective heat loss, and evaporation rates, explaining the observed trends in productivity.
2. Consistent performance profile:
  The results show that the rate of increase in productivity is similar across all wind speed trials, indicating that the system’s performance profile remains consistent. This suggests that the enhanced convective cooling provided by higher wind speeds has a predictable and linear impact on productivity.
  The consistent performance profile also highlights the robustness of the integrated SS-MD design, as it can effectively utilize higher wind speeds to improve productivity without significant changes in system behavior, as captured by Eq. (12).
3. Role of wind speed in overall performance:
  Wind speed is a critical performance indicator for the SS section, as it directly influences the evaporation rate and thermal efficiency of the system. The results demonstrate that higher wind speeds lead to higher productivity, validating the importance of considering local wind conditions in the design and operation of solar desalination systems.

The findings from Fig. 13 demonstrate that wind speed significantly impacts the overall distillate flux of the integrated SS-MD system. Higher wind speeds enhance convective cooling in the SS section, increasing evaporation rates and improving productivity. These results are consistent with the heat transfer principles and the permeability equation, highlighting the importance of optimizing wind conditions in the design of solar desalination systems.

A comparison of the distillate production performance of this work with some promising passive solar still designs from previous literature is presented in Table 2. The proposed integrated SS-MD system demonstrates superior productivity under optimal conditions, validating its potential for efficient solar desalination.

**Table 2:** Distillate production comparison with previous literature.

Study	System	Productivity ${\text{L}}/{\text{m}}^{2} \;{\text{day}}$	Key findings
Sathaumurthy et al. ^43^	Standalone SS single basin	4.6	Limited by low thermal efficiency
Kabeel et al. ^16^	Pyramid solar still coated with TiO2 nano black paint	6.6	Relatively high productivity with a low cost per liter
Singh ^17^	Solar Still with evacuated tube collector and external reflector	8.1	Sophisticated system, requires maintainability
Shmroukh and Ookawara ^9^	Standalone SS multi basin with internal reflectors	8.285	Limited by bottom basin wall thermal losses
Proposed system	Integrated SS-MD	46.94	High productivity due to heat recovery (June, ten-stage)

## Conclusions and recommendations

This study investigates the performance of an integrated solar still with membrane distillation through a comprehensive numerical analysis. The key findings are summarized as follows:

- *Effect of MD stages* Increasing the number of MD stages significantly enhances distillate productivity, with the ten-stage configuration achieving a productivity of 47 ${\text{L}}/{\text{m}}^{2} \;{\text{day}}$ This represents a remarkable improvement, approximately ten times higher than the output of an unmodified solar still under identical conditions and about five times greater than the highest production rate reported in the literature.
- *Effect of brine depth* Lowering the brine depth maximizes the productivity by optimizing thermal energy utilization. Although it is challenging to keep the depth at 0.5 mm, it gives an improvement in the productivity 50% higher than 1 mm depth and about 100% of 1.5 mm
- *Seasonal variations* The system performs best under June conditions (high solar intensity and longer daytime), with productivity decreasing in March and December due to lower solar flux and shorter daylight hours, surpassing December’s distillate productivity values by more than double.
- *Wind speed impact* Higher wind speeds enhance convective cooling in the SS section, increasing productivity by 3–5% for every 2 m/s increase in wind speed.

The present results are promising to that encourages further investigations on this integration The following future research directions are recommended:

- *Hybrid energy sources* This includes the implementation of indirect feed heating with solar water heating systems (evacuated tube or flat plate) with the possibility of adopting the photovoltaic panels to power the entire SS-MD module. In other words, evaluating the active integration of SS-MD module which would improve the overall system efficiency and the sustainability goals in providing both water and energy to rural areas.
- *Experimental validation* Conduct pilot-scale experiments to validate the numerical model and optimize system design aiming at designing a medium to large scale modules suitable for rural and arid coastal areas.
- *Economic and environmental analysis* Perform a cost–benefit analysis and life cycle assessment to evaluate the economic viability and environmental impact of the proposed system.

## Data Availability

All data generated or analysed during this study are included in this published article.

## Abbreviations

${\text{AGMD}}$: Air-gap membrane distillation
${\text{DCMD}}$: Direct contact membrane distillation
${\text{FPC}}$: Flat plate collector
${\text{ISS}}$: Improved stepped solar still
$${\text{MD}}$$: Membrane distillation
$${\text{PTFE}}$$: Polytetrafluoroethylene
${\text{SGMD}}$: Sweeping gas membrane distillation
$${\text{SS}}$$: Solar still
${\text{SSS}}$: Stepped Solar still
${\text{VMD}}$: Vacuum membrane distillation

## List of symbols

$$A$$: Area (m^2^)
$$A_{side}$$: Side area (m^2^)
$$a$$: Activity
$C_{p}$: Specific heat capacity (kJ/kg K)
$D_{wa}$: Diffusion coefficient of water vapor in air (m^2^/s)
$$d$$: Layer thickness (m)
$$Gr$$: Grashof number
$h$: Heat transfer coefficient (W/m^2^ C)
$h_{b}$: Heat transfer coefficient from basin to water (W/m^2^ C)
$h_{fg}$: Latent heat of vaporization (J/kg)
$h_{ti}$: Total heat transfer coefficient in the solar still (W/m^2^ C)
$h_{to}$: Total heat transfer coefficient outside the solar still (W/m^2^ C)
$$I$$: Intensity (W/m^2^)
$J$: Total membrane distillation flux (W/m^2^)
$K$: Permeability coefficient (kg/m^2^ s Pa)
$$k$$: Thermal conductivity (W/m C)
$M$: Molar mass (kg/mol)
$M_{s}$: Mass of distillate produced by solar still (kg)
$n$: Number of MD stages
$P$: Pressure/vapor pressure (Pa)
$$Pr$$: Prandtl number
$$Q$$: Heat energy (W)
$r$: Membrane pore radius (m)
$$t$$: Time
$$T$$: Temperature (°C)
$$W_{s}$$: Wind speed (m/s)
$$Y$$: Mass fraction

## Subscript

$$a$$: Ambient
$$Al$$: Aluminum layer
$air$: Air gap layer
$$b$$: Basin layer
$$bm$$: Base metal
$$c$$: Condensing layer
$c,ba$: Convection from basin to air
$c,ga$: Convection from glass to air
$c,wg$: Convection from water to glass
$$e$$: Evaporating layer
$e,wg$: Evaporation from water to glass
$$g$$: Glass
$$i$$: Time step
$$ins$$: Insulation layer
$j$: MD stage number
$$m$$: Membrane layer
$r,ga$: Radiation from glass to air
$r,wg$: Radiation from water to glass
$$s$$: Solar
$$sp$$: Spacer
$$sky$$: Sky temperature
$$to$$: Total external
$$ti$$: Total internal
$$v$$: Vapor
$ve$: Vapor in evaporating layer
$vc$: Vapor in condensing layer
$$w$$: Water

## Greek letters

$$\alpha$$: Absorptivity
$$\varepsilon$$: Emissivity
$$\epsilon$$: Average porosity
$\sigma$: Stefan Boltzmann constant
$$\tau$$: Tortuosity
